# Myelin Basic Protein Post-Translational Modifications Orchestrate Astrocyte Regulatory Networks

**DOI:** 10.3390/neurosci7010026

**Published:** 2026-02-13

**Authors:** Jeremy Ramsden, Marika Chikviladze, Nino Mamulashvili, Lali Shanshiashvili, David Mikeladze

**Affiliations:** 1 Department of Biomedical Research, The University of Buckingham, Hunter Street, Buckingham MK18 1EG, UK; j.ramsden@colbas.org; 2 Centre for Molecular Recognition, Collegium Basilea, 4053 Basel, Switzerland; 3 Institute of Chemical Biology, Ilia State University, 45 Ilia Chavchavadze Avenue, 0179 Tbilisi, Georgiadavit_mikeladze@iliauni.edu.ge (D.M.); 4 Ivane Beritashvili Centre of Experimental Biomedicine, 0162 Tbilisi, Georgia

**Keywords:** astrocytes, myelin basic protein, inflammation, demyelination, cytokines, liver X receptor

## Abstract

Multiple sclerosis (MS) pathogenesis involves not only immune-mediated myelin injury but also glial responses. We examined how three charge isomers of myelin basic protein (MBP)—native (C1), phosphorylated (C4), and citrullinated (C8)—modulate rat astrocytes. Cytokines were quantified and grouped (pro/anti-inflammatory, chemotactic, neurotrophic, angiogenic, tissue remodeling), and regulatory markers assessed. C1 strongly upregulated the lipid-sensing receptor LXR, and reduced global DNA methylation; C4 moderately enhanced LXR; C8 failed to activate LXR or alter methylation. Functionally, C1 attenuated IL-1β, IL-6 and GM-CSF while increasing IL-10 and certain chemokines. C4 elicited an intermediate pattern, inducing CX3CL1 (fractalkine), CCL20, VEGF-A and TIMP-1 with minor effects on classical cytokines. In contrast, C8 triggered a robust pro-inflammatory phenotype, increasing IL-1α/β, TNF-α and GM-CSF, with higher IL-10, fractalkine, CCL20, VEGF-A and TIMP-1. All isomers suppressed IFN-γ, IL-4 and CNTF. These data indicate that MBP post-translational modifications drive distinct astrocyte phenotypes through integrated cytokine, metabolic and epigenetic pathways: C1 favors immune regulation and repair, C4 blends inflammatory and reparative cues, and C8 amplifies neuroinflammation. Understanding how modified MBP shapes astrocyte behavior provides mechanistic insight into lesion evolution in MS and suggests astrocyte-directed strategies to modulate neuroinflammation and promote remyelination.

## 1. Introduction

Astrocytes are integral participants in the central nervous system (CNS) immune milieu, actively responding to injury and demyelination by secreting a broad array of cytokines and chemokines. In multiple sclerosis (MS) and its animal model, experimental autoimmune encephalomyelitis (EAE), reactive astrocytes exhibit a Janus-faced role—they can produce pro-inflammatory mediators that drive immune cell recruitment and tissue damage, yet also release anti-inflammatory and neurotrophic factors that support repair [1,2,3]. Understanding the factors that tip this balance is critical. One such factor is myelin basic protein (MBP) [4], a major myelin antigen that becomes exposed during demyelination. Fragments of MBP are released into the extracellular space in active MS lesions and may form damage-associated molecular patterns, engaging astrocytes and microglia [5]. Importantly, MBP exists in multiple charge isomeric forms due to extensive post-translational modifications (PTMs), including phosphorylation and citrullination (deimination). Under physiological conditions, the unmodified C1 isoform predominates, whereas modified isoforms—particularly citrullinated MBP (C8)—are present at low levels but become relatively enriched in pathological contexts associated with demyelination and neuroinflammation [5]. These modifications alter MBP’s structure and charge, potentially changing how glial cells recognize and respond to myelin debris [6].

Citrullination of MBP—conversion of positively charged arginine to neutral citrulline residues—is markedly elevated in MS lesions [7]. Hypercitrullinated MBP (often referred to as the C8 isomer in charge-focusing nomenclature) is structurally less compact and exposes immunodominant epitopes [8]. Prior studies have shown that citrullinated MBP is highly immunogenic and pro-inflammatory: it can elicit innate immune responses, priming astrocytes and macrophages toward inflammatory phenotypes [9]. Moscarello et al. first proposed in 2007 that deiminated MBP in MS lesions “enhances the ability of astrocytes to release proinflammatory mediators”, linking this MBP isomer to chronic lesion inflammation [10]. More recently, we demonstrated that the citrullinated C8 isomer triggers nuclear factor-κB activation in astrocytes, inducing the secretion of pro-inflammatory molecules (e.g., HMGB1, NO and IL-17A) whereas unmodified MBP did not [9]. These findings align with evidence that citrullinated myelin actively inhibits repair: in a demyelinated mouse brain slice model, injection of citrullinated myelin (rich in C8 MBP) provoked microglial tumor necrosis factor-α (TNF-α) production and impaired remyelination, an effect reversed by TNF-α neutralization [7]. Thus, citrullination of MBP is hypothesized to exacerbate neuroinflammation and hinder myelin repair in MS.

Phosphorylation is another common MBP modification, though its role in MS pathology is less understood. MBP can be phosphorylated by various kinases, potentially altering its charge (i.e., adding negative charges) and interactions. Some studies suggest MBP phosphorylation might reduce its membrane-binding affinity and could influence antigenicity [5]. The MBP C4 isomer examined here represents a phosphorylated form of MBP. By comparing it with C1 (unmodified MBP) and C8 (citrullinated MBP), we aim to delineate how specific MBP modifications influence astrocyte behavior, not only through cytokine production but also via LXR signaling and DNA methylation dynamics.

Astrocytes actively shape the CNS inflammatory environment. When stimulated by factors like interleukin-1β (IL-1β) or TNF-α, astrocytes upregulate chemokines and cytokines that attract leukocytes into the CNS. For example, astrocytic CXCL12 (SDF-1α) expression is induced by IL-1β and by soluble MBP, promoting recruitment of T cells and monocytes to lesions [11], and incubation of astrocytes with C8 induces IL-33 expression [12]. Similarly, CCL2 (MCP-1) and CCL20 (MIP-3α) produced by reactive astrocytes help recruit monocytes and Th17 cells, respectively, amplifying the inflammatory loop. Astrocytes also produce cytokines like IL-6 and GM-CSF that activate microglia and support pathogenic Th17 responses in EAE [13]. Indeed, GM-CSF is now recognized as a key mediator in EAE/MS, driving microglial activation and monocyte entry; blocking GM-CSF or its receptor ameliorates disease in models [14]. On the other hand, astrocytes can secrete anti-inflammatory and neurotrophic factors—e.g., IL-10, TGF-β, CNTF, NGF—which limit inflammation and support neurons and oligodendrocytes [1]. The net outcome of astrocyte activation in demyelination depends on the balance of these factors. Therefore, if different forms of myelin debris skew astrocytes towards distinct cytokine profiles, this could profoundly influence lesion progression: a predominantly pro-inflammatory astrocyte response would promote immune-mediated damage, whereas an anti-inflammatory/trophic response might facilitate resolution and remyelination [13].

Beyond cytokine signaling, astrocytic responses to MBP are also shaped by nuclear receptor and epigenetic pathways. Liver X receptors (LXRs) act as cholesterol sensors that couple lipid metabolism with inflammatory gene regulation, and their activation in astrocytes has been shown to suppress NF-κB-driven cytokine expression while supporting remyelination [15]. Protein arginine deiminase 2 (PAD2), the main enzyme mediating citrullination of MBP, is upregulated in MS lesions and contributes to structural destabilization of myelin proteins [16]. In addition, dynamic changes in DNA methylation control astrocyte gene expression under injury, enabling fate transitions such as stem-like reprogramming [17].

In this study, we systematically analyze how primary astrocytes respond to three MBP charge isomers—C1, native/unmodified; C4, phosphorylated; and C8, citrullinated—in terms of cytokine and chemokine expression. We present experimental data profiling a panel of cytokines secreted by astrocytes after exposure to each MBP isomer, and we group these cytokines by functional roles—pro-inflammatory, anti-inflammatory, chemotactic, neurotrophic, angiogenic, tissue remodeling—for interpretation. Our goal is to integrate these findings with current knowledge of cytokine function in MS/EAE, thereby elucidating how MBP modifications may differentially influence astrocyte-mediated inflammation and myelination. We hypothesize that citrullinated MBP C8 induces a more inflammatory astrocyte profile than native MBP C1, consistent with C8’s known immunogenicity, and that phosphorylated MBP C4 may produce an intermediate or distinct pattern of astrocyte activation. Understanding these differences could shed light on the mechanisms by which biochemical changes in myelin contribute to either detrimental inflammation or repair processes in demyelinating diseases.

## 2. Materials and Methods

### 2.1. Animal Sourcing and Euthanasia

Primary astrocytes were obtained from 2-day-old Wistar rat pups. All procedures involving animals were conducted in accordance with institutional and national guidelines for the care and use of laboratory animals and were approved by the local Institutional Animal Care Bioethics Commission (meeting protocol N 5/20.08.2025).

Neonatal rats were sourced from the Institute’s breeding colony. They were maintained under standard housing conditions (a 12 h light/dark cycle, controlled temperature and humidity, with unrestricted access to food and water).

For cell isolation, pups were humanely euthanized by decapitation using sterile surgical scissors, a method widely accepted for neonatal rodents according to international ethical guidelines. Immediately after euthanasia, brains were removed under sterile conditions and used for primary astrocyte isolation.

### 2.2. Primary Astrocyte Cultures

Primary cortical astrocytes were prepared from neonatal rat brains (postnatal day 1–2 Sprague–Dawley rats) using standard procedures. Briefly, dissociated cortical cells were plated on poly-D-lysine (sc-136156, Santa Cruz Biotechnology, Inc., Dallas, TX, USA)-coated culture surfaces and grown in DMEM/F12 (Gibco, Bleiswijk, The Netherlands) medium with 10% FBS (Sigma-Aldrich Co., St Louis, MO, USA) and antibiotics. After reaching confluence (~2–3 weeks), cultures were shaken to remove microglia and oligodendrocytes, which poorly adhere to poly-D-lysine, yielding astrocyte-enriched monolayers. Adherent astrocytes were then trypsinized (Carbosynth, Compton, Berkshire, UK) and replated for experiments at a suitable density (typically 50,000–100,000 cells/well in 24-well plates). All procedures were conducted in serum-free medium 24 h prior to and during stimulation to eliminate exogenous factors. Astrocyte purity was routinely monitored by morphological assessment using phase-contrast light microscopy (Jenco Instruments, San Diego, CA, USA), revealing the characteristic stellate morphology of astrocytes and the absence of cells with microglial or oligodendroglial appearance.

### 2.3. MBP Isomers and Treatments

MBP charge isomers C1, C4, and C8 were isolated from bovine CNS myelin by cation-exchange chromatography and preparative isoelectric focusing, as described in previous work [5]. C1 corresponds to the most basic (least modified) MBP isoform, C8 to the most acidic (extensively citrullinated) isoform, and C4 is an intermediate isoform characterized by MBP with one or more phosphorylations (and possibly minor deamidation) but minimal citrullination. Purified MBP isomers were verified by SDS-PAGE (Bio-Rad Laboratories, Hercules, CA, USA) and stored lyophilized until use. For treatments, astrocytes were incubated with 0.5 μM of either C1, C4 or C8 MBP for 24 h at 37 °C (control cells were incubated under identical conditions without MBP). This concentration and timepoint were chosen based on preliminary dose–response tests and literature indicating astrocyte responses to MBP concentrations in the submicromolar range [9]. Triplicate wells were used for each condition in each experiment.

### 2.4. Cytokine Profiling

After 24 h of exposure to MBP charge isomers, cell culture supernatants were collected and cleared by centrifugation. For cytokine profiling, supernatants obtained from three independent astrocyte isolations were pooled to ensure sufficient sample volume and enable simultaneous quantification of multiple analytes. Cytokine and chemokine levels were measured using a Rat Cytokine Array C1(8) kit (RayBiotech, Norcross, GA, USA), according to the manufacturer’s instructions.

The analytes quantified included: IL-1α, IL-1β, IL-4, IL-6, IL-10, TNF-α, IFN-γ (classical cytokines); GM-CSF (growth factor/cytokine); chemokines CINC-1, CINC-2, CINC-3, CXCL5 (LIX), CCL2 (MCP-1), CCL20 (MIP-3α), and CX3CL1 (fractalkine); neurotrophic factors CNTF and β-NGF; VEGF-A (angiogenic factor); and TIMP-1 (tissue inhibitor of metalloproteinases-1), measured as a marker of tissue remodeling response.

Cytokine array signals were detected by chemiluminescence and quantified by densitometric analysis using the manufacturer-provided analysis software. Each pooled sample was analyzed in two technical replicates. Signal intensities were background-subtracted and normalized to internal positive controls included on each membrane, according to the assay protocol. Normalized values were expressed relative to untreated control samples for subsequent statistical analysis.

### 2.5. Western Blotting

Following incubation in the presence or absence of MBP charge isomers, astrocytes were subjected to western blotting (electrophoresis and blotting apparatus were from Bio-Rad Laboratories, Hercules, CA, USA).

The following primary antibody was used: anti-LXRα/β (G-10) (Santa Cruz Biotechnology, Dallas, TX, USA). Immunoreactivity was detected by enhanced chemiluminescence autoradiography (ECL kit, Santa Cruz Biotechnology, Dallas, TX, USA). Protein concentrations were determined using a BCA protein assay kit (Thermo Fisher Scientific, Rockford, IL, USA). Signal quantification from western blots was assisted by Image Studio software version 5.2.5 (LI-COR Biotech LLC, Lincoln, NE, USA).

### 2.6. DNA Extraction and PCR

Genomic DNA was extracted from primary astrocytes using the GF-1 Tissue DNA Extraction kit (Vivantis Technologies, Subang Jaya, Selangor, Malaysia, Cat# GF-TD-050) according to the manufacturer’s instructions. PCR amplification was carried out using Taq DNA Polymerase with Standard Taq Buffer (New England Biolabs, Ipswich, MA, USA).

Global DNA methylation analysis was performed using the EZ DNA Methylation kit (Zymo Research, Irvine, CA, USA), which includes bisulfite conversion of genomic DNA. PCR amplification was carried out with the following parameters: 95 °C for 40 s; 35 cycles of denaturing at 95 °C for 40 s, annealing at 59 °C for 45 s, extension at 72 °C for 45 s; and a final incubation at 68 °C for 5 min. Specific primer pairs were: forward—GAGCCAGGCTAGACGGA; reverse—GCTCCCGCAGCATCTTT (expected amplicon size: 211 bp).

### 2.7. Data Analysis

Cytokine levels in MBP-treated astrocytes are expressed relative to control (untreated) cells. Statistical analysis was performed using one-way ANOVA followed by Dunnett’s multiple-comparisons test, with each MBP-treated group compared a priori to the control.

For western blot and qPCR analyses, data are presented as mean ± SEM from at least three independent biological experiments.

For cytokine profiling, data are presented as mean ± SD derived from duplicate technical replicates obtained from pooled culture supernatants of three independent astrocyte isolations.

A value of *p* < 0.05 was considered statistically significant.

## 3. Results

**Figure 1:**
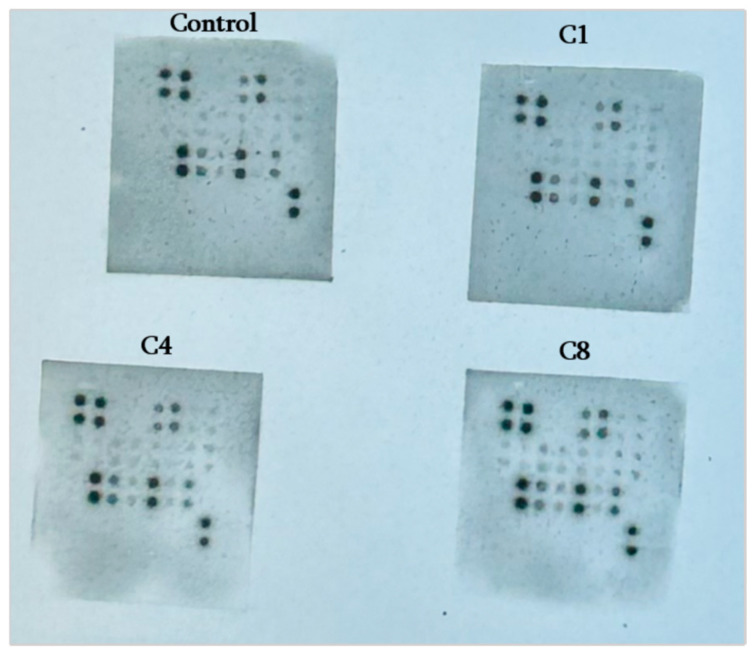
Rat cytokine array of astrocytes untreated and treated with MBP charge isomers C1, C4 and C8. These representative array membranes show cytokine expression patterns under control, C1 (native MBP), C4 (phosphorylated MBP) and C8 (citrullinated MBP) conditions. Each dot represents a duplicated antibody spot corresponding to the specific cytokines or chemokines provided by the kit maker.

Overview of astrocyte cytokine responses to MBP isomers: Astrocytes exposed to MBP underwent significant changes in their cytokine secretion profile, which differed markedly depending on the MBP isomer: control cells vs. native C1, phosphorylated C4, or citrullinated C8-treated cells (Figure 1, Figure S1: Suppl.-AAR-CYT-1_Analysis_To ASTR).

For each cytokine, we noted whether MBP treatment caused a significant increase, decrease, or no significant change vs. the control (untreated). These outcomes were then categorized by the known biological function of each cytokine (pro-inflammatory, anti-inflammatory, chemotactic, etc.). 1. Pro-inflammatory cytokines were defined as those that typically promote or sustain immune responses: IL-1α, IL-1β, TNF-α, IFN-γ, IL-6 and GM-CSF; 2. Anti-inflammatory cytokines included those that dampen immune responses or promote Th2/regulatory profiles: IL-10 and IL-4; 3. Neutrophil-attracting CXC chemokines: CINC-2, CINC-3 and LIX; 4. Monocyte/macrophage- and lymphocyte-attracting chemokines: chemokines that recruit leukocytes, CC chemokines like CCL2, CCL20, as well as fractalkine; 5. Neurotrophic and growth factors included CNTF and β-NGF, which support neuronal and oligodendroglial survival; VEGF-A, a key vascular endothelial growth factor; TIMP-1, which modulates breakdown of the extracellular matrix. Data are presented in tables and heatmaps to summarize directional changes. All experiments were repeated at least twice to check reproducibility.

For clarity, we first summarize these findings in Table 1 before detailing each functional category. The table provides a global view; each cytokine is listed (rows) with an arrow indicating whether its level was increased (↑), decreased (↓) or unchanged (–) compared to the untreated control, under each MBP isomer (columns). As can be seen, C1 (native MBP) predominantly caused downward modulation of inflammatory cytokines, whereas C8 (citrullinated MBP) led to widespread increases in multiple cytokines, and C4 (phospho-MBP) showed a mix of minimal or moderate changes. The table emphasizes how post-translational modifications of MBP selectively modulate astrocyte inflammatory and homeostatic signaling, but all three isomers shared some effects in common (e.g., all decreased CINC-3 and CNTF), but differed in others (e.g., only C8 increased TNF-α).

### 3.1. Pro-Inflammatory Cytokines

**Figure 2:**
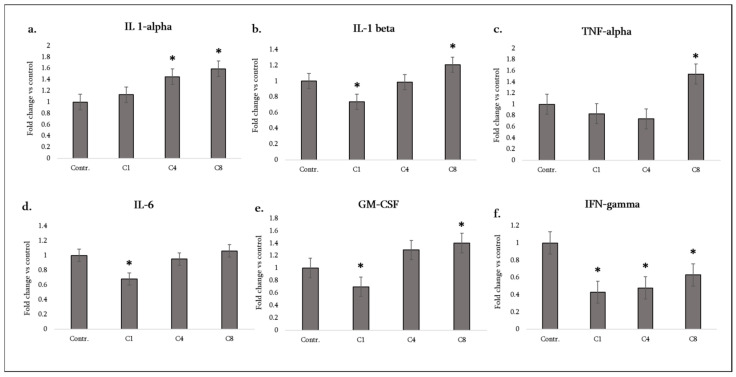
Pro-inflammatory cytokine production by astrocytes following MBP treatment. Production of IL-1α (**a**), IL-1β (**b**), TNF-α (**c**), IL-6 (**d**), GM-CSF (**e**), and IFN-γ (**f**) was measured in the culture medium of control astrocytes (labeled Contr.) and astrocytes treated with MBP charge isomers (C1, C4, or C8). Cytokine levels were assessed using a rat cytokine antibody array and quantified by densitometric analysis. Data are expressed as fold change relative to the untreated control (set to 1) and represent mean ± SD derived from duplicate technical replicates of pooled supernatants obtained from three independent astrocyte isolations. Statistical analysis was carried out using one-way ANOVA followed by Dunnett’s multiple-comparisons test (each MBP-treated group vs. control). * signifies *p* < 0.05 vs. control.

Astrocytes are not the primary source of IL-1α, IL-1β, or TNF-α in the CNS under homeostatic conditions, but they can produce these cytokines when activated and thereby amplify neuroinflammation [18]. We saw from our experiments that the MBP isomers have divergent effects on astrocytic IL-1 and TNF expression. C1, unmodified MBP, significantly suppressed IL-1β production (to below control levels) and did not induce TNF-α or IL-1α (these remained at baseline, showing no change vs. controls) (Figure 2a–c). C4, phosphorylated MBP, strongly upregulated IL-1α secretion by astrocytes (Figure 2a), whilst having no significant effect on IL-1β or TNF-α (both remained at control levels) (Figure 2b,c). C8, citrullinated MBP, provoked the most prominent response: astrocytes treated with C8 significantly increased secretion of IL-1α, IL-1β and TNF-α compared to the control (Figure 2a–c).

Notably, IL-1β—a key driver of the inflammasome-mediated inflammation—was increased by C8 (whereas C4 caused no change, and C1 decreased it). IL-1α, an alarmin often released by dying cells or activated glia, was increased by both C4 and C8, with C8 showing a larger effect. TNF-α, a central mediator of oligodendrocyte damage and blood–brain barrier disruption, was only increased under C8 treatment (C1 and C4 did not significantly change TNF-α levels). These results indicate that citrullinated MBP triggers astrocytes to adopt a highly pro-inflammatory cytokine profile, whereas native MBP dampens certain pro-inflammatory outputs (especially IL-1β) and phosphorylated MBP has a selective pro-inflammatory effect (IL-1α) without broad activation of other cytokines like TNF-α.

Another important pro-inflammatory cytokine is IFN-γ (interferon-gamma), typically produced by T cells and NK cells, but also measurable at low levels in glia. All three MBP isomers caused a decrease in detectable IFN-γ levels from astrocyte cultures (Figure 2f). While astrocytes are not major producers of IFN-γ, this result suggests MBP exposure might somehow decrease any baseline IFN-γ (possibly by consuming it or by suppressing IFN-γ-inducing signals). In any case, IFN-γ changes likely play a minor role here given its low basal expression in astrocytes.

IL-6 and GM-CSF are two additional cytokines highly relevant in MS. IL-6 is a pleiotropic cytokine that promotes Th17 differentiation and acute phase responses, and GM-CSF (granulocyte-macrophage colony-stimulating factor) is a key molecule driving microglial activation and monocyte recruitment in the CNS. C1 caused a significant decrease in astrocyte IL-6 secretion (approximately halving it vs. control), whereas C4 and C8 caused no significant change in IL-6 (astrocyte IL-6 remained at control levels under these treatments) (Figure 2d). For GM-CSF, C1 again had a suppressive effect (GM-CSF decreased in C1-treated astrocytes), C4 showed only a slight upward trend (increase) that did not reach statistical significance, and C8 induced a clear increase in astrocyte-derived GM-CSF (Figure 2e). Thus, only the citrullinated MBP drove significant GM-CSF release. This is noteworthy because astrocyte-derived GM-CSF can strongly activate microglia and peripheral myeloid cells; our data imply that C8 could foster a more pathogenic feed-forward loop in EAE/MS by this mechanism. In contrast, native MBP (C1) may actually decrease astrocytic GM-CSF output, potentially limiting CNS inflammation.

Overall, the pro-inflammatory cytokine profile can be summarized as follows: C1 (native)—broadly down-regulatory (decreasing IL-1β, IL-6, GM-CSF, with no TNF induction); C4 (phospho)—mildly pro-inflammatory (increasing IL-1α only, otherwise neutral); C8 (citrulline)—strongly pro-inflammatory (increasing IL-1α, IL-1β, TNF-α, GM-CSF). These differences highlight how MBP modifications influence the intensity of the astrocytic inflammatory response. We note that IL-1 and GM-CSF are crucial for driving Th17-cell mediated inflammation in EAE, and TNF-α contributes to oligodendrocyte injury, suggesting that C8’s ability to induce these factors could exacerbate demyelinating disease, whereas C1’s suppressive effect might be associated with a more controlled or resolving inflammatory environment.

### 3.2. Anti-Inflammatory Cytokines

**Figure 3:**
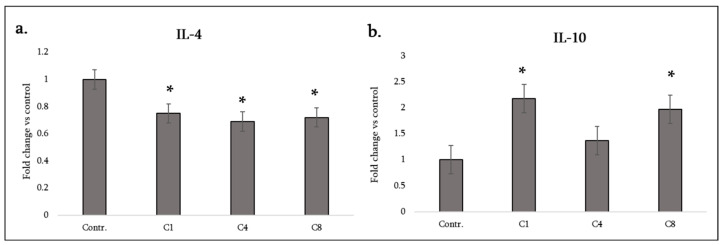
Anti-inflammatory cytokine production by astrocytes following MBP treatment. Production of IL-4 (**a**) and IL-10 (**b**) was measured in the culture medium of control astrocytes (labeled Contr.) and astrocytes treated with MBP charge isomers (C1, C4, or C8). Cytokine levels were assessed using a rat cytokine antibody array and quantified by densitometric analysis. Data are expressed as fold change relative to the untreated control (set to 1) and represent mean ± SD derived from duplicate technical replicates of pooled supernatants obtained from three independent astrocyte isolations. Statistical analysis was carried out using one-way ANOVA followed by Dunnett’s multiple-comparisons test (each MBP-treated group vs. control). * signifies *p* < 0.05 vs. control.

We examined two cytokines associated with anti-inflammatory or Th2/regulatory responses, IL-10 and IL-4. IL-10 is a potent anti-inflammatory cytokine that can be produced by astrocytes and microglia to suppress excessive inflammation and promote repair. IL-4 is a Th2 cytokine that in the CNS can promote alternative activation of glia and has anti-inflammatory effects (IL-4 suppresses production of IL-1 and TNF while enhancing certain growth factors). However, astrocytes themselves do not typically produce IL-4 in notable amounts. In our cultures, IL-4 was barely detectable in controls, and all MBP treatments led to a small but consistent decrease (Figure 3a), which may not be biologically significant given the low baseline. Essentially, none of the MBP isomers induced IL-4 production in astrocytes (as expected, since IL-4 is usually produced by lymphocytes, not glia).

Interestingly, our data show that both C1 and C8 induced a significant increase in IL-10 secretion from astrocytes, whereas C4 did not affect it (Figure 3b).

In summary, IL-10 secretion was enhanced by C1 and C8, highlighting that even the strongly pro-inflammatory C8 drives some anti-inflammatory output (perhaps via NF-κB signaling also inducing IL-10 as a negative feedback effect). C4’s lack of IL-10 induction is notable—it suggests that phosphorylated MBP triggers pro-inflammatory cues (like IL-1α) without concomitant induction of anti-inflammatory dampening, potentially skewing astrocytes to a more reactive state. Table 1 (lower half) summarizes these findings along with those concerning neurotrophic factors, as will be described next. Astrocyte-derived chemokines orchestrate the recruitment of various immune cells to sites of CNS injury. We group the measured chemokines by the type of immune cells they attract.

### 3.3. Neutrophil-Attracting CXC Chemokines

**Figure 4:**
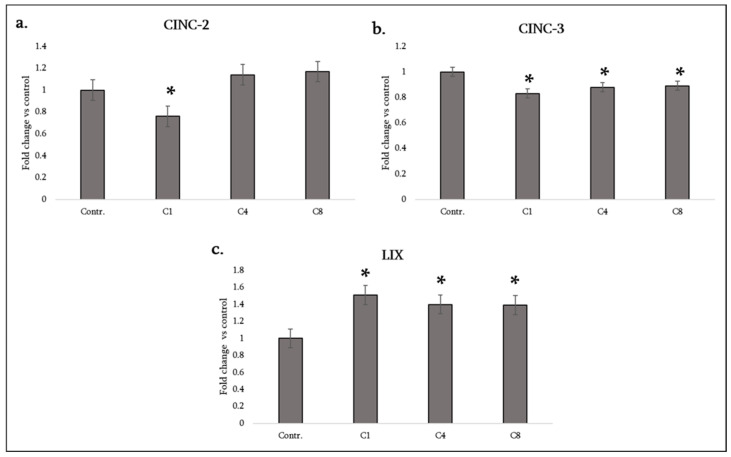
Neutrophil-attracting chemokine production by astrocytes following MBP treatment. Levels of CINC-2 (**a**), CINC-3 (**b**), and LIX/CXCL5 (**c**) were measured in the culture medium of control astrocytes (labeled Contr.) and astrocytes treated with MBP charge isomers (C1, C4, or C8). Chemokine levels were assessed using a rat cytokine antibody array and quantified by densitometric analysis. Data are expressed as fold change relative to the untreated control (set to 1) and represent mean ± SD derived from duplicate technical replicates of pooled supernatants obtained from three independent astrocyte isolations. Statistical analysis was carried out using one-way ANOVA followed by Dunnett’s multiple-comparisons test (each MBP-treated group vs. control). * signifies *p* < 0.05 vs. control.

CINC-2, CINC-3, and LIX: These ELR^+^ CXC chemokines are functional rodent homologues of human CXCL1/2/3 and CXCL5, and play a central role in recruiting and activating neutrophils at sites of CNS injury or inflammation. Their regulation provides important insight into how astrocytes modulate acute innate immune cell infiltration. In rodents, CINC-1, CINC-2 and CINC-3 (cytokine-induced neutrophil chemoattractants) are functional analogs of human CXCL1/CXCL2/CXCL3 (e.g., IL-8 homologues), and LIX (CXCL5) also attracts neutrophils, which can contribute to acute tissue damage in fulminant neuroinflammation. These chemoattractants play a central role in recruiting and activating neutrophils at sites of CNS injury or inflammation. Their regulation provides important insight into how astrocytes modulate acute innate immune cell infiltration. In our astrocyte cultures, baseline production of these CXC chemokines was low, but some changes were observed. C1 (native MBP) significantly decreased CINC-2 levels (CXCL2) while C4 and C8 caused no change in CINC-2 (remaining at baseline) (Figure 4a). For CINC-3 (CXCL3), all three isomers (C1, C4, C8) caused a decrease in its level (Figure 4b). The decrease in neutrophil chemoattractant level by C1 might indicate an anti-inflammatory stance (it leads to less neutrophil recruitment), whereas even C8 did not increase the levels of these chemokines, implying that neutrophil recruitment might not be a dominant feature of astrocyte response to MBP (perhaps the recruitment is handled by microglia). In contrast, LIX (CXCL5) was significantly increased by all three isomers—astrocytes treated with C1, C4 or C8 each showed increased LIX compared to the control (Figure 4c).

LIX is another neutrophil chemokine; its concurrent upregulation suggests that astrocytes may preferentially use CXCL5 (rather than members of the CINC family) to signal neutrophils. The reasons for this differential effect (CINC-2/CINC-3 down, but CXCL5 up) are unclear but may relate to different regulatory pathways for ELR-positive CXC chemokines. Regardless, the net effect on neutrophil recruitment is mixed; MBP exposure could both inhibit and stimulate different neutrophil chemoattractants. The increase in LIX (CXCL5) especially under C8 and C4 might support some neutrophil presence in chronic lesions, although neutrophils are less prominent than monocytes in MS.

### 3.4. Monocyte/Macrophage- and Lymphocyte-Attracting Chemokines

**Figure 5:**
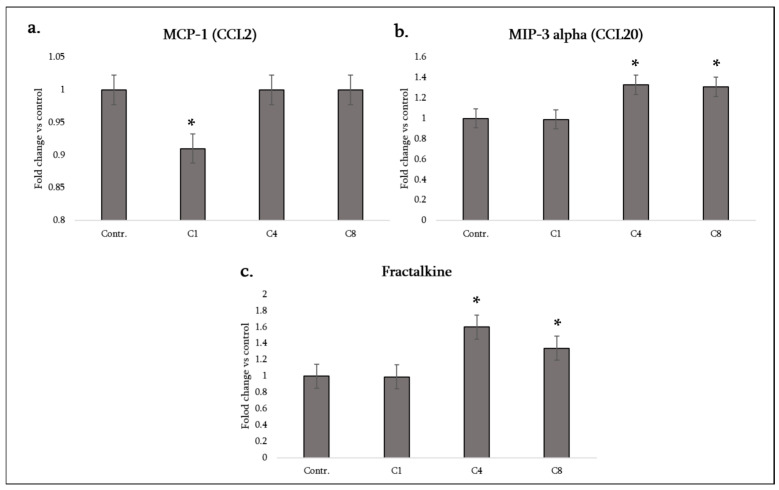
Monocyte/macrophage- and lymphocyte-attracting chemokine production by astrocytes following MBP treatment. Levels of MCP-1/CCL2 (**a**), MIP-3α/CCL20 (**b**), and fractalkine/CX3CL1 (**c**) were analyzed in the culture medium of control astrocytes (labeled Contr.) and astrocytes treated with MBP charge isomers (C1, C4, or C8). Chemokine levels were assessed using a rat cytokine antibody array and quantified by densitometric analysis. Data are expressed as fold change relative to the untreated control (set to 1) and represent mean ± SD derived from duplicate technical replicates of pooled supernatants obtained from three independent astrocyte isolations. Statistical analysis was carried out using one-way ANOVA followed by Dunnett’s multiple-comparisons test (each MBP-treated group vs. control). * signifies *p* < 0.05 vs. control.

CCL2 (MCP-1) attracts monocytes and CCR2+ T cells and is a major chemokine in MS lesions, driving peripheral immune cell entry. We found that C1 significantly decreased CCL2 (MCP-1) secretion by astrocytes, whereas C4 and C8 did not change CCL2 (remaining at control levels). Thus, native MBP may quell a key monocyte-recruiting signal from astrocytes, potentially diminishing immune cell infiltration, while modified MBP did not suppress it (though, notably, nor did they increase it). On the other hand, CCL20 (MIP-3α)—a chemokine that selectively attracts CCR6+ cells such as Th17 lymphocytes and dendritic cells—was strongly induced by both C4 and C8, but not by C1 (Figure 5a).

Astrocytes normally produce little CCL20, but under C4 or C8 exposure MIP-3α levels rose significantly. This is an important finding: phosphorylated and citrullinated MBP stimulated astrocytes to secrete CCL20, which could preferentially recruit pathogenic Th17 cells into the CNS, since Th17 cells characteristically express CCR6 and migrate towards CCL20. Indeed, in EAE, CCL20 expression in the CNS is required for Th17 infiltration. C1 (native MBP) did not provoke any CCL20 increase (it remained at baseline in C1-treated cultures) (Figure 5b). Therefore, the modified forms of MBP seem to encourage astrocytes to recruit adaptive immune cells more than the native form does.

Another intriguing chemokine is CX3CL1 (fractalkine). Uniquely, fractalkine exists as both a membrane-bound adhesion molecule and a soluble chemokine; it primarily signals through CX3CR1 on microglia, monocytes and NK cells. Neurons usually express fractalkine, but astrocytes generally do not under normal conditions. However, our data show that C4 and C8 treatments lead to a significant increase in fractalkine (CX3CL1) release by astrocytes, whereas C1 had no effect (fractalkine remained undetected or at the baseline with C1) (Figure 5c). This suggests that both phosphorylated and citrullinated MBP can induce astrocytes to produce fractalkine, potentially as a signal to modulate microglia.

Fractalkine has a complex role; it can attract CX3CR1+ monocytes/microglia, but it can also serve as a neuron–glia communication signal that diminishes microglial activation when membrane-bound. Soluble fractalkine from astrocytes might help recruit microglia to sites of myelin debris or alter their activation state. The fact that C1 did not induce fractalkine whereas C4/C8 did is another indication that modified MBP elicits a “reactive” astrocyte phenotype involving neuron–microglia signaling molecules.

In summary, astrocyte chemokine responses differed according to the MBP isomer; C1 tended to decrease certain chemokines (CINC-2, CINC-3, CCL2) and increased only LIX, whereas C4, and especially C8, increased several chemokines (CX3CL1, CCL20, LIX) without suppressing others. Notably, C8 combined a high pro-inflammatory cytokine response with induction of chemokines that recruit both innate (monocytes, microglia via fractalkine) and adaptive (Th17 via CCL20) immune cells. C4 induced a similar pattern for chemokines (fractalkine and CCL20 up), albeit with less pro-inflammatory cytokine output. Table 1 summarizes the changes in key chemokines under each condition.

### 3.5. Neurotrophic and Growth Factors

Astrocytes play a supportive role for neurons and oligodendrocytes by supplying growth factors. We measured CNTF and β-NGF as representatives of neurotrophic factors, and VEGF-A as an angiogenic factor relevant for lesion vascularization and potentially oligodendrocyte precursor cell support. Additionally, TIMP-1 was assessed as a factor related to tissue remodeling and protection (inhibiting matrix metalloproteinases that can degrade the extracellular matrix and blood–brain barrier).

**Figure 6:**
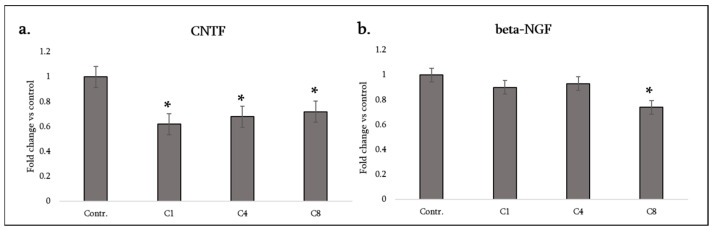
Neurotrophic factor production by astrocytes following MBP treatment. Protein expression of CNTF (**a**) and β-NGF (**b**) was assessed in the culture medium of control astrocytes (labeled Contr.) and astrocytes treated with MBP charge isomers (C1, C4, or C8). Neurotrophic factor levels were assessed using a rat cytokine array and quantified by densitometric analysis. Data are expressed as fold change relative to the untreated control (set to 1) and represent mean ± SD derived from duplicate technical replicates of pooled supernatants obtained from three independent astrocyte isolations. Statistical analysis was carried out using one-way ANOVA followed by Dunnett’s multiple-comparisons test (each MBP-treated group vs. control). * signifies *p* < 0.05 vs. control.

CNTF (ciliary neurotrophic factor): Astrocytes are a major source of CNTF in the CNS, especially upon injury, and CNTF promotes oligodendrocyte survival and remyelination. Strikingly, all three MBP isomers caused a decrease in CNTF secretion from astrocytes. C1, C4 and C8 each significantly decreased CNTF levels relative to the control (Figure 6a). This uniform response suggests that exposure to myelin debris, regardless of its modification, may signal astrocytes to downregulate CNTF production. The loss of this neuroprotective factor could have implications; during demyelination, lower CNTF might diminish support for oligodendrocytes and neurons. Notably, even C1 (otherwise relatively anti-inflammatory) caused CNTF to decrease, indicating that this effect may be mediated by a common receptor or pathway triggered by MBP itself (e.g., LRP1-mediated uptake of MBP might suppress CNTF expression in astrocytes, although the mechanism is unclear). In the MS context, decreased astrocytic CNTF upon myelin damage could limit the natural repair attempt.

β-NGF (beta nerve growth factor): NGF is another trophic factor (more associated with neuron survival and immune modulation). Astrocytes can produce NGF at low levels. In our experiments C8 (citrullinated MBP) caused a significant decrease in NGF, whereas C1 and C4 had no effect (NGF remained similar to the control for C1 and C4) (Figure 6b). Thus, citrullinated MBP uniquely diminished NGF availability. Lowered NGF might adversely affect neurons or oligodendrocyte precursor cells in lesions. This finding is consistent with C8’s generally detrimental profile—it not only increases inflammatory cytokines but also diminishes growth factors that could help recovery.

**Figure 7:**
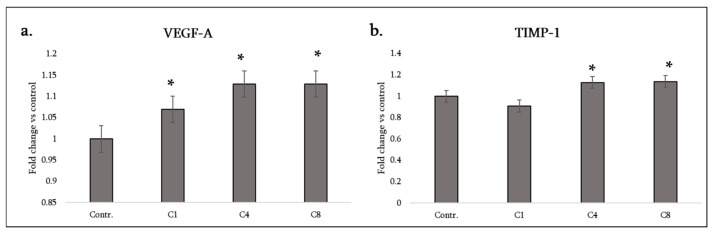
Angiogenic and extracellular matrix–modulating factor production by astrocytes following MBP treatment. Levels of VEGF-A (**a**) and TIMP-1 (**b**) were measured in the culture medium of control astrocytes (labeled Contr.) and astrocytes treated with MBP charge isomers (C1, C4, or C8). Protein levels were assessed using a rat cytokine array and quantified by densitometric analysis. Data are expressed as fold change relative to the untreated control (set to 1) and represent mean ± SD derived from duplicate technical replicates of pooled supernatants obtained from three independent astrocyte isolations. Statistical analysis was carried out using one-way ANOVA followed by Dunnett’s multiple-comparisons test (each MBP-treated group vs. control). * signifies *p* < 0.05 vs. control.

VEGF-A (vascular endothelial growth factor-A): Astrocyte-derived VEGF-A can influence angiogenesis and blood–brain barrier integrity in lesions. Interestingly, all three MBP isomers stimulated a significant increase in VEGF-A secretion from astrocytes, with C4 and C8 showing especially strong increases (in the data, C4 and C8 show greater VEGF induction than C1) (Figure 7a). This suggests that astrocytes, when encountering myelin debris (modified or not), may promote angiogenic responses—possibly to restore blood supply or due to hypoxia signaling in lesions. VEGF can be double-edged; it supports new vessel growth (which might aid lesion repair), but excessive VEGF can increase vascular permeability, exacerbating inflammation. In MS, elevated VEGF in active lesions has been reported, and it may correlate with blood vessel proliferation at the lesion edge. Our results imply that MBP fragments could be a trigger for astrocytic VEGF release in demyelinating areas. The fact that C4 and C8 induced more VEGF than C1 might mean that modified MBP (especially when citrullinated or phosphorylated) is a stronger stimulus for angiogenesis. This could relate to greater activation of transcription factors like HIF-1α or NF-κB by those isomers.

TIMP-1 (tissue inhibitor of metalloproteinases-1): TIMP-1 is produced by astrocytes to counteract matrix metalloproteinases (MMPs) that degrade extracellular matrix and tight junctions. High TIMP-1 can help stabilize the blood–brain barrier and extracellular environment, potentially aiding remyelination by protecting nascent myelin and promoting a scar matrix. We found that C4 and C8 significantly increased TIMP-1 release, whereas C1 did not change TIMP-1 (Table 1). Thus, both phosphorylated and citrullinated MBP induce astrocytes to upregulate this tissue remodeling inhibitor. This could be viewed as a protective response: when there is inflammatory damage, astrocytes under C4/C8 influence might attempt to curtail MMP activity (for instance, MMP-9 is known to contribute to myelin and BBB damage in MS; TIMP-1 would inhibit MMP-9) (Figure 7b).

C1’s lack of effect on TIMP-1 suggests native MBP does not trigger this aspect of the reactive astrocyte program, whereas the modified isomers do, potentially indicating that injury-associated forms of MBP (phosphorylated from cell stress or citrullinated from inflammation) signal astrocytes to initiate wound-healing mechanisms like TIMP-1 upregulation.

Collectively, in the neurotrophic and repair category, C8 appears most detrimental (it decreases both CNTF and NGF, depriving the CNS of key neurotrophic support, though it increases VEGF and TIMP-1), C4 has a mixed effect (lower CNTF but higher VEGF and TIMP-1), and C1’s effects might be considered milder (it does lower CNTF, which is detrimental, but raises VEGF somewhat and does not alter TIMP-1 or NGF). It is noteworthy that all forms lead to CNTF loss, meaning astrocytes encountering any myelin material may transiently shift away from neuroprotective mode. This could be one reason why areas of active demyelination have impaired support for neurons/oligodendrocytes. On the other hand, the increase in VEGF and TIMP-1, especially with C4 and C8, indicates astrocytes are also engaging in damage response to maintain or restore the lesional environment (angiogenesis and matrix preservation).

### 3.6. LXR Expression in Astrocytes

**Figure 8:**
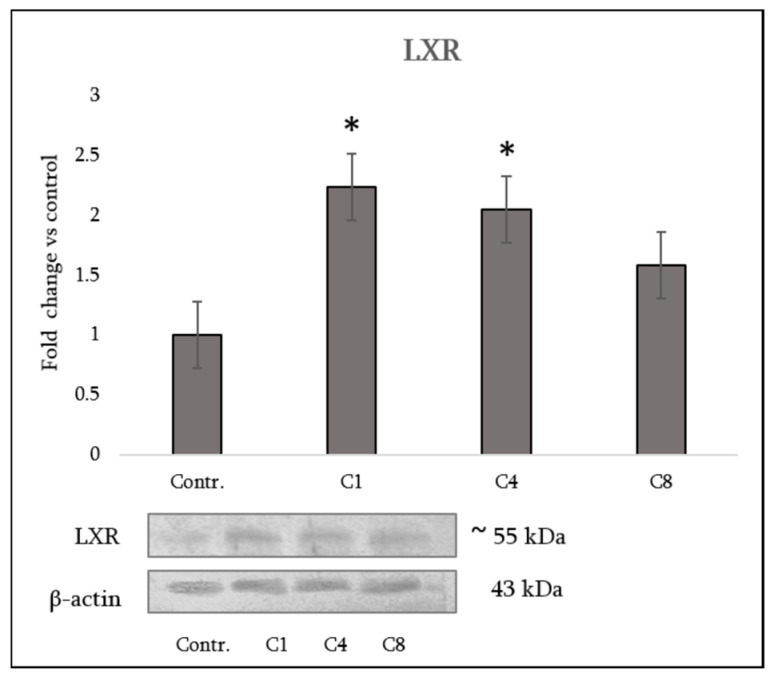
LXR expression in primary astrocytes following exposure to MBP charge isomers. Representative western blot showing LXR protein levels in control astrocytes (labeled Contr.) and astrocytes treated with MBP isomers C1 (native), C4 (phosphorylated), and C8 (citrullinated). Densitometric analysis demonstrates increased LXR expression following C1 treatment, a moderate increase with C4, and no statistically significant change with C8 compared to control. Data are presented as means ± SEM from *n* = 3 independent biological experiments. Statistical analysis used one-way ANOVA followed by Dunnett’s multiple-comparisons test (each MBP-treated group vs. control). * signifies *p* < 0.05 vs. control.

LXRs are key regulators of lipid homeostasis. They also have notable anti-inflammatory effects, especially in macrophages. They can inhibit pro-inflammatory genes and are essential in conditions like metabolic disorders [16]. Western blot analysis demonstrated differential regulation of LXR by MBP charge isomers. The native C1 isomer induced the most pronounced effect, with expression levels increased nearly 2.5-fold compared with control astrocytes. Phosphorylated C4 also caused an increase in LXR expression, though to a lesser extent. The citrullinated C8 isomer produced only a modest increase, which did not reach statistical significance (Figure 8, Figure S8: WB-Suppl. for Figure 8; WB-For Figure 8 (LXR) densitometry; WB-For Figure 8(1); WB-beta-actin- For Figure 8).

### 3.7. Global DNA Methylation in Astrocytes

**Figure 9:**
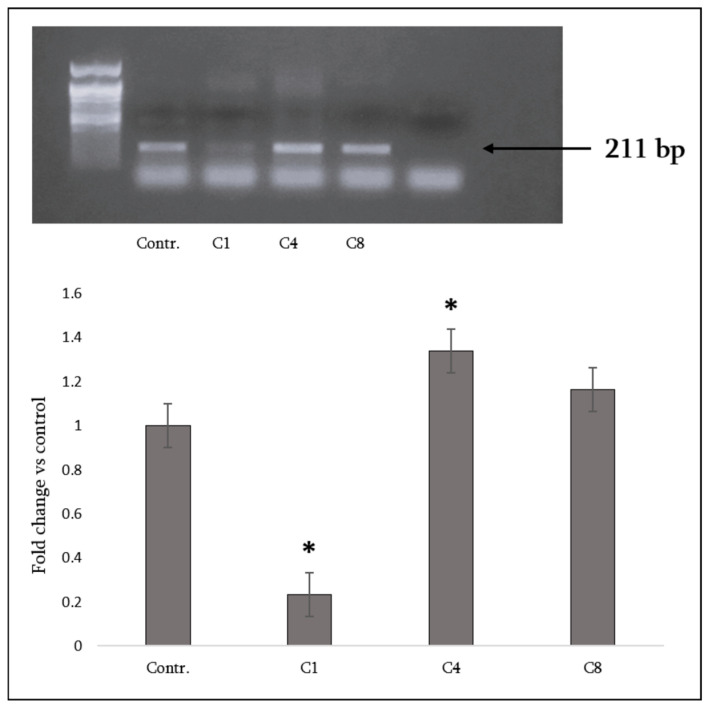
Global DNA methylation analysis in primary rat astrocytes exposed to MBP charge isomers. (**Top**) Representative PCR-based methylation assay showing amplicons corresponding to methylated CpG content in control astrocytes (labeled Contr.) and astrocytes treated with MBP isomers C1, C4, and C8; on the extreme right is water as a negative control. Band intensities were quantified by densitometry. (**Bottom**) Quantification of global DNA methylation expressed as -fold change relative to the untreated control. Data are presented as means ± SEM from *n* = 3 independent biological experiments. Statistical analysis was carried out using one-way ANOVA followed by Dunnett’s multiple-comparisons test (each MBP-treated group vs. control). * signifies *p* < 0.05 vs. control.

Epigenetic regulation affects cytokine release from macrophages through mechanisms like DNA methylation. Hypermethylation of various promoters in macrophages can suppress the expression of negative regulators of cytokine signaling, resulting in the overproduction of inflammatory cytokines such as TNF-α and IL-6 [19]. To assess whether different MBP isomers influence cytokine production via epigenetic regulation, DNA methylation levels were examined. Assessment of global DNA methylation revealed distinct effects of MBP isomers. C1 decreased methylation levels by approximately 3–4-fold relative to control astrocytes. In contrast, C4 increased methylation, while C8 did not produce significant changes, although a tendency toward increase was observed (Figure 9, Figure S9: PCR-Suppl. for Figure 9(1); PCR-Suppl. for Figure 9(2); PCR-Suppl. for Figure 9(3)).

## 4. Discussion

Our findings demonstrate that primary astrocytes mount distinct cytokine responses depending on the biochemical form of the MBP they encounter. Native MBP (C1) induces a relatively modulatory or dampened inflammatory response in astrocytes, characterized by decreased production of key pro-inflammatory mediators (IL-1β, IL-6, GM-CSF) and an increase in the anti-inflammatory cytokine IL-10. In contrast, citrullinated MBP (C8) provoked astrocytes to adopt a markedly pro-inflammatory and chemotactic phenotype, with significant secretions of IL-1α, IL-1β, TNF-α, and GM-CSF, as well as of chemokines CCL20 and fractalkine, which can recruit pathogenic immune cells. Phosphorylated MBP (C4) elicited an intermediate response: it shared some features with C8 (induction of fractalkine, CCL20, TIMP-1, VEGF) but did not trigger broad pro-inflammatory cytokine release (notably, TNF-α remained unchanged and IL-1β was not increased under C4). These differential profiles have important implications for neuroinflammation and myelin repair in diseases like MS.

### 4.1. Astrocyte Activation by Modified MBP

Our results support and extend previous studies suggesting that post-translational modifications of MBP act as molecular switches for glial activation. The pronounced pro-inflammatory response to C8 concurs with previously reported results identifying citrullinated MBP as a driver of astroglial and microglial inflammation [9]. Mechanistically, deiminated MBP has been shown to activate NF-κB signaling in astrocytes [9], which would explain the upregulation of NF-κB-dependent cytokines we observed (IL-1, IL-6, TNF, GM-CSF, IL-10 are all NF-κB-responsive gene products). Indeed, Shanshiashvili et al. previously reported that only C8 (and not C1) activated astrocyte NF-κB and induced release of HMGB1, NO and IL-17A [9]; while IL-17A was not included in our present cytokine panel, their finding correlates with our observation that C8 strongly induces IL-1β and GM-CSF—two cytokines that can stimulate Th17 cells and possibly astrocytic IL-17 production as a secondary effect [9]. The increase in IL-10 alongside IL-1 and TNF in C8-treated astrocytes might reflect an attempted counterregulation via NF-κB’s ability to co-induce IL-10, or activation of other transcription factors like PPAR-γ that promote IL-10. Nonetheless, the net balance under C8 is tipped towards inflammation, consistent with C8’s known immunogenicity in MS lesions [10].

In contrast, the anti-inflammatory bias under C1 (native MBP) suggests that unmodified myelin debris may not strongly trigger astrocytes or might even engage receptors that suppress activation. One could speculate that intact MBP, being more cationic, might bind differently to astrocyte receptors or be taken up more efficiently (reducing surface Toll-like receptor engagement). MBP has been reported to interact with astrocyte LRP1 (a receptor involved in phagocytosis of myelin) [9]. Efficient clearance of MBP via LRP1 might lead to an anti-inflammatory outcome, as uptake of debris can sometimes trigger PPAR-γ pathways that suppress pro-inflammatory genes [9]. Interestingly, Shanshiashvili et al. found that C1 enhanced M2 polarization markers in macrophages, whereas C8 enhanced M1 [9]. By analogy, our data suggest C1 could be nudging astrocytes toward a less reactive (potentially “A2” reparative) state, whereas C8 pushes them toward an “A1” neurotoxic reactive state [20]. In our case, C8 indeed induced the archetypal A1 cytokines IL-1β and TNF-α, and decreased trophic factors CNTF/NGF, matching an A1-like profile. C1’s induction of IL-10 and reduction in pro-inflammatory factors aligns more with an A2-like or at least non-A1 profile. Therefore, MBP citrullination may directly contribute to formation of neurotoxic astrocytes in MS lesions, whereas normal MBP might not.

### 4.2. Recruitment of Immune Cells

The differential chemokine expression we observed has implications for which immune cells are drawn into the CNS. Under C8, increased CCL20 would specifically favor infiltration of CCR6+ Th17 cells, which are known to be highly encephalitogenic in EAE (they produce IL-17, GM-CSF, etc., driving inflammation). In fact, IL-1β and IL-6 (both elevated by C8 in astrocytes) are required for Th17 differentiation; thus C8-stimulated astrocytes could not only recruit Th17 cells via CCL20 but also create an environment (with IL-1β, IL-6, and GM-CSF) that sustains and reactivates these Th17 cells in the CNS [7,11]. Meanwhile, fractalkine upregulation by C8 and C4 suggests enhanced crosstalk with microglia. Fractalkine signaling (CX3CL1–CX3CR1) can have neuroprotective effects by keeping microglia in a surveillant state; however, it can also serve as a chemoattractant in its soluble form. In EAE, deficiency in CX3CR1 (fractalkine receptor) has been shown to sometimes worsen disease by unleashing uncontrolled microglial activation, implying that fractalkine from astrocytes or neurons often acts as a brake on microglia activity. Thus, the induction of fractalkine by C4 and C8 could be interpreted as a protective attempt by astrocytes to restrain microglia that are activated by the ongoing inflammation. This is a nuanced point: although C8 makes astrocytes highly inflammatory, the concurrent rise in fractalkine and IL-10 could reflect the astrocytes’ built-in regulatory feedback to prevent runaway damage. It is conceivable that in chronic lesions, astrocytes exposed to a lot of citrullinated myelin produce fractalkine to engage CX3CR1 on microglia, which might limit microglial neurotoxicity (CX3CR1 signaling tends to suppress microglial production of cytokines like IL-1 and TNF). The interplay of signals is complex; whether this compensatory effect is sufficient is doubtful, given that MS lesions do progress.

For phosphorylated MBP (C4), the astrocyte response seems tailored more towards immune recruitment and repair, rather than direct inflammation. C4 did not elevate TNF or IL-1β, but it did induce IL-1α (which can stimulate neighboring cells and initiate inflammatory responses in a more limited fashion) and chemokines fractalkine/CCL20. C4 also induced TIMP-1 and VEGF, suggesting an astrocyte phenotype geared towards moderating tissue damage and supporting angiogenesis. One interpretation is that phosphorylation of MBP, which might occur during early myelin injury or stress, signals astrocytes that damage is occurring but perhaps not in full autoimmune mode, prompting them to fortify the environment (via TIMP-1 to protect the matrix, and VEGF to secure blood supply) and alert the immune system in a controlled way (via IL-1α and CCL20 to attract specific immune cells). The “C4 astrocyte” could thus represent a state of partial reactivity, straddling both protective and pro-inflammatory actions.

Implications for MS lesions: MS lesion stages could differentially contain MBP isomers—acute early damage might release mostly native or minimally modified MBP, whereas chronic active lesions accumulate citrullinated MBP (due to prolonged PAD enzyme activity) [21]. Our data predict that early demyelination (native MBP exposure) would cause astrocytes to secrete factors like IL-10 and moderate amounts of VEGF, perhaps restraining the initial inflammation to some extent. In contrast, in chronic active lesions where MBP is heavily citrullinated, astrocytes would be driven into a pro-inflammatory state, perpetuating the lesion’s inflammatory milieu through IL-1β, TNF-α and GM-CSF. This self-perpetuating inflammation can result in a failure of lesion resolution and impede remyelination. Notably, TNF-α from astrocytes (and microglia) can induce oligodendrocyte death via TNF-R1 signaling and also disturb the blood–brain barrier. The presence of citrullinated MBP has been correlated with areas of most severe myelin destruction and lymphocyte infiltration in both MS and EAE [22]. Our results provide mechanistic insight: citrullinated MBP drives astrocytes to secrete exactly those factors (TNF, IL-1, GM-CSF) that amplify immune-mediated myelin damage. Moreover, the decrease in CNTF and NGF by C8 would deprive oligodendrocytes of survival signals, aggravating demyelination and slowing remyelination.

Conversely, the astrocytic IL-10 increase we observed with C1 (and also with C8) might reflect an in vivo phenomenon in certain lesion contexts. The increase from native MBP (C1) treatment suggests that unmodified myelin debris might trigger an astrocytic feedback mechanism to counteract inflammation. The increase from citrullinated MBP (C8) treatment possibly reflects an attempt by astrocytes to restrain the strong pro-inflammatory activation caused by C8. Phosphorylated MBP (C4), despite inducing some inflammatory factors, did not induce IL-10. IL-10 is known to be protective in EAE; astrocyte-targeted IL-10 expression can lessen EAE severity by dampening microglial activation and fostering an environment conducive to repair [23]. Intriguingly, both the least modified and most modified MBP isoforms induced IL-10 in astrocytes, indicating that regardless of the initial astrocyte response, IL-10 is eventually produced as a countermeasure. In acute lesions (with C1), IL-10 might help to swiftly resolve inflammation. In chronic lesions (with C8), the IL-10 might not be able to overcome the simultaneous surge of pro-inflammatory cytokines—essentially a failing compensatory mechanism, or one that arrives with a delay relative to the pro-inflammatory cascade, rendering it relatively ineffectual.

### 4.3. Relevance to Remyelination

Remyelination in MS can be impeded by inflammatory cytokines and supported by growth factors. Astrocyte-derived TNF-α and IL-1β have been implicated in inhibiting the differentiation of oligodendrocyte precursor cells (OPCs) into myelinating oligodendrocytes. IL-1β, for instance, can drive astrogliosis and a toxic environment, whereas neutralizing IL-1β in vivo can improve remyelination in some models. GM-CSF can indirectly inhibit remyelination by maintaining microglia in an inflammatory state rather than a regenerative state. On the other hand, CNTF and IL-10 promote OPC survival and maturation. Our data indicate that astrocytes in the presence of citrullinated MBP are likely to engender an environment unfavorable for remyelination: high IL-1β, TNF, and GM-CSF, coupled with low CNTF and NGF. Indeed, a recent study in a toxin-induced demyelination model showed that injection of citrullinated myelin debris prevented normal remyelination, an effect attributed to TNF-α from microglia.

It stands to reason that astrocyte cytokines (like those we observed under C8) would synergize with microglial factors to block remyelination. In contrast, astrocytes encountering primarily native MBP (e.g., in cases of milder injury) would secrete more CNTF, more IL-10, and less TNF—conditions that are more permissive for oligodendrocyte regeneration and myelin repair. This suggests a model where the biochemical state of myelin debris influences whether a lesion will be inflammatory: chronic (citrullinated MBP driving astrocytes to an inflammatory phenotype) or more benign and repair-friendly (native MBP causing a restrained astrocyte response).

These findings highlight potential therapeutic targets. If citrullinated MBP sustains astrocyte-mediated inflammation, lessening protein citrullination (e.g., via PAD inhibition) may mitigate chronic inflammatory cascades in progressive MS [8]. Enhancing astrocyte and microglial clearance of myelin debris—such as through LRP1-mediated phagocytosis or early macrophage recruitment—could also prevent debris accumulation and its harmful post-translational modifications. Notably, this implies phosphorylated MBP (C4)-induced TIMP-1 and VEGF, factors that may help stabilize lesion microenvironments. Astrocyte-derived TIMP-1 is particularly relevant, as it supports remyelination by inhibiting MMP-driven barriers to OPC migration. Thus, astrocyte activation is not uniformly detrimental; the challenge is to enhance protective signals (TIMP-1, IL-10, CNTF) while limiting pro-inflammatory outputs (TNF, IL-1, GM-CSF). Our data indicate that astrocyte responses can be “tuned” by the biochemical state of myelin debris. Approaches that influence debris recognition—such as modulating TLR pathways or opsonin—may therefore bias astrocytes towards repair even in the presence of modified myelin.

EAE studies show that loss of astrocytes can worsen disease by removing key regulatory functions [13], emphasizing astrocytes’ dual protective and pathogenic roles. Our results lead to a refinement of this description by indicating that the astrocyte phenotype depends on the MBP form encountered; unmodified MBP elicits protective responses, whereas citrullinated MBP promotes damaging ones. It would be useful if further work identified receptors and pathways differentiating these responses. Citrullinated MBP may preferentially activate PRRs (e.g., TLR2/4), while phosphorylated MBP may engage MAPK cascades. MBP has been proposed to signal through integrins and TLRs as a damage-associated molecular pattern (DAMP); clarifying the mechanisms would guide selective targeting of harmful astrocyte outputs while preserving beneficial functions.

A key rationale for assessing LXR expression with cytokines lies in the dual role of LXRs in lipid metabolism and immune regulation. Astrocytes are central to CNS cholesterol trafficking for myelin synthesis [24]. LXR activation promotes cholesterol turnover, apolipoprotein production and myelin gene expression, while suppressing NF-κB-driven cytokines [25,26]. LXR/RXR agonists attenuate glial inflammation and support remyelination [15,22]. Evaluating LXR status therefore provided a mechanistic link between MBP isomer-induced cytokine changes and alterations in lipid pathways essential for myelin maintenance.

We found that the native, highly cationic C1 isomer produced the strongest astrocytic response, increasing LXR expression nearly 2.5-fold and suppressing IL-1β, IL-6 and IFN-γ while elevating IL-10. This aligns with the literature showing that LXR activation enhances remyelination and oligodendrocyte support [16,25]. Collectively, these data suggest that C1 promotes a protective, pro-remyelinating astrocytic phenotype in which LXR signaling is a central regulatory driver [22].

Phosphorylated C4 induced a moderate increase in LXR and an intermediate cytokine pattern, consistent with partial preservation of MBP’s regulatory capacity [27]. In contrast, citrullinated C8 failed to activate LXR and produced a strongly pro-inflammatory profile (IL-1α, IL-1β, TNF-α) while reducing neurotrophic factors (CNTF, NGF). This mirrors states in which impaired LXR signaling compromises myelin lipid recycling and remyelination [27,28]. The combination of weak LXR activity and dominant inflammatory signaling underscores the pathogenic impact of extensive MBP citrullination [29]. Given that LXR/RXR pathways regulate cholesterol homeostasis and inflammatory cascades in MS [30], diminished LXR responsiveness in C8-treated astrocytes further supports its disease-relevant maladaptive profile.

Astrocytic responses were examined using purified MBP charge isomers rather than unseparated total MBP preparations, which would mimic the actual biological environment in living organisms. Purified MBP charge isomers allow isomer-specific effects to be resolved but cannot reveal potential interactions within heterogeneous MBP mixtures.

Although key regulatory pathways were validated in the present study, further validation of selected cytokine responses at the protein or transcript level remains an important direction for future studies.

Overall, these findings demonstrate that MBP post-translational modifications differentially shape astrocyte function by modulating the LXR–cytokine axis. C1 maintains a homeostatic, pro-remyelinating environment through strong LXR induction and anti-inflammatory cytokine expression, whereas C8 drives inflammatory, lipid-dysregulated states associated with impaired repair. This scenario concurs with recent reviews highlighting LXR signaling as a therapeutic target in demyelinating and neurodegenerative disorders [15,30].

Our PCR analysis revealed that MBP isomers also modulate global DNA methylation in astrocytes, providing additional mechanistic insights. Analysis of DNA methylation patterns revealed that C1 markedly reduced methylation levels (3–4-fold decrease), while C4 increased methylation and C8 showed no significant changes, albeit with a tendency toward elevation. These divergent epigenetic responses indicate that MBP isomers can influence astrocytic gene regulation not only through cytokine and nuclear receptor pathways but also by modulating epigenetic mechanisms. Moreover, recent findings demonstrate that astrocyte stem-like conversion under stress or injury is mediated by dynamic DNA methylation changes, further supporting the functional relevance of our observed MBP isomer-induced methylation modulation in astrocytes. In particular, the hypomethylating effect of C1 may facilitate the expression of genes supporting anti-inflammatory and pro-remyelinating functions [17], whereas the hypermethylating effect of C4 could contribute to stress-related or maladaptive transcriptional profiles [31]. The lack of strong methylation changes in response to C8 lends further weight to the view that its pathogenic impact in astrocytes is more closely linked to inflammatory signaling and diminished neurotrophic support than to direct epigenetic regulation.

## 5. Conclusions

In this study, a comprehensive experimental investigation and analysis of how three charge isomers of myelin basic protein influence the cytokine milieu and regulatory pathways of primary astrocytes.

- Native MBP (C1) promoted an anti-inflammatory and pro-remyelinating phenotype, with increased IL-10, strong LXR upregulation and markedly decreased DNA methylation;
- Phosphorylated MBP (C4) elicited a mixed response, combining moderate LXR with increased methylation and partial induction of repair-associated factors;
- Citrullinated MBP (C8) drove a pro-inflammatory, neurotoxic profile with impaired LXR activation, stable methylation and loss of neurotrophic support.

Together, these findings demonstrate that MBP modifications shape astrocyte function not only through cytokine secretion but also via lipid-sensing and epigenetic mechanisms.

Biochemical modifications of MBP act as molecular switches that determine whether astrocytes promote regeneration or drive chronic inflammation, highlighting novel targets for therapeutic modulation in demyelinating disease.

## Figures and Tables

**Table 1:** Summary of statistically significant changes and observed trends in astrocyte cytokine responses to MBP charge isomers. Arrows indicate statistically significant changes compared to the untreated control (↑ increased, ↓ decreased; *p* < 0.05), whereas “–” denotes no statistically significant change. Non-significant directional changes are reported as trends and interpreted cautiously in the text. Cytokines are grouped according to their primary functional categories. The table summarizes distinct cytokine response patterns induced by unmodified MBP (C1), phosphorylated MBP (C4), and citrullinated MBP (C8) in astrocytes. Full quantitative cytokine expression values (mean ± SD, derived from duplicate technical replicates prior to normalization) are provided in Supplementary Table S1.

Cytokine (Synonyms)	Functional Group	C1	C4	C8
CINC-2 (CXCL2)	Chemokine (pro-inflammatory)	↓	–	–
CINC-3 (CXCL3/MIP-2)	Chemokine (pro-inflammatory)	↓	↓	↓
CNTF (Ciliary neurotrophic factor)	Neurotrophic cytokine	↓	↓	↓
Fractalkine (CX3CL1)	*Chemotactic* factor (atypical CX3C)	–	↑	↑
GM-CSF (CSF2)	Pro-inflammatory cytokine/myeloid activator	↓	–	↑
IFN-γ	Pro-inflammatory cytokine	↓	↓	↓
IL-1α	Pro-inflammatory cytokine(alarmin)	–	↑	↑
IL-1β	Pro-inflammatory cytokine	↓	–	↑
IL-4	Anti-inflammatory (Th2 cytokine)	↓	↓	↓
IL-6	Pro-inflammatory cytokine/Th17	↓	–	–
IL-10	Anti-inflammatory cytokine	↑	–	↑
LIX (CXCL5)	Chemokine (pro-inflammatory)	↑	↑	↑
MCP-1 (CCL2)	*Chemotactic* factor (pro-inflammatory)	↓	–	–
MIP-3α (CCL20)	*Chemotactic* factor (pro-inflammatory)	–	↑	↑
β-NGF	Neurotrophic factor	–	–	↓
TIMP-1	Tissue remodeling factor	–	↑	↑
TNF-α	Pro-inflammatory cytokine/cytotoxic	–	–	↑
VEGF-A	Angiogenic factor	↑	↑	↑

## Data Availability

All data generated or analyzed during this study are included in this published article and its Supplementary Materials.

## Supplementary Materials

The following supporting information can be downloaded at: https://www.mdpi.com/article/10.3390/neurosci7010026/s1, Figure S1: Suppl.-AAR-CYT-1_Analysis_To ASTR. Figure S8: WB-Suppl. for Figure 8; WB-For Figure 8 (LXR) densitometry; WB-For Figure 8(1); WB-beta-actin- for Figure 8. Figure S9: PCR-Suppl. for Figure 9(1); PCR-Suppl. for Figure 9(2); PCR-Suppl. for Figure 9(3); Table S1: Quantitative cytokine expression profile in primary astrocytes treated with MBP charge isomers.

## Abbreviations

CINC	Cytokine-induced neutrophil chemoattractants
CNS	Central nervous system
CNTF	Ciliary neurotrophic factor
CX3CR1	Fractalkine receptor
EAE	Model experimental autoimmune encephalomyelitis
GM-CSF	Granulocyte-macrophage colony-stimulating factor
IFN-γ	Interferon-gamma
IL-1β	Interleukin-1β
LXRs	Liver X receptors
MBP	Myelin basic protein
MS	Multiple sclerosis
NF-κB	Nuclear factor-kappa B
OPCs	Oligodendrocyte precursor cells
PTMs	Post-translational modifications
TIMP-1	Tissue Inhibitor of Metalloproteinases-1
TNF-α	Tumor necrosis factor-α
VEGF-A	Vascular endothelial growth factor-A
β-NGF	beta nerve growth factor
