# Supplementary figures and images for: Myelin Basic Protein Post-Translational Modifications Orchestrate Astrocyte Regulatory Networks
Source: NeuroSci. 2026 Feb 13;7(1):26. doi: 10.3390/neurosci7010026 (PMC12921786; doi:10.3390/neurosci7010026)

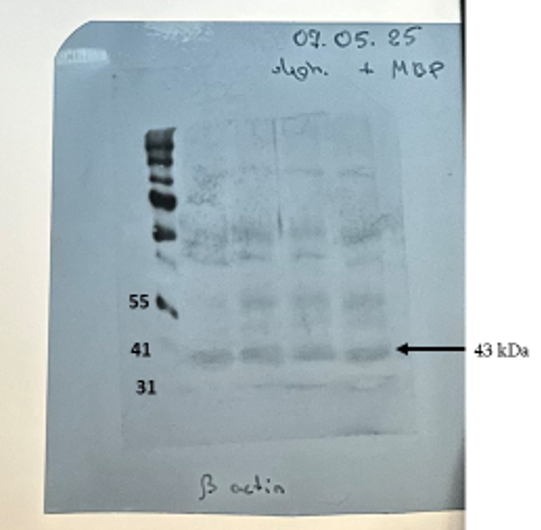

Supplement: Supplementary file 1 [file neurosci-07-00026-s001.zip › Figure S8 WB-beta-actin- For Figure 8.tif]

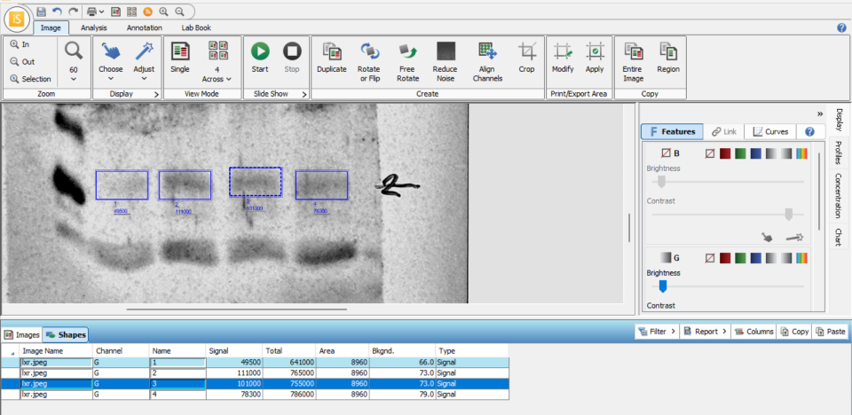

Supplement: Supplementary file 1 [file neurosci-07-00026-s001.zip › Figure S8 WB-For Figure 8 (1).tif]

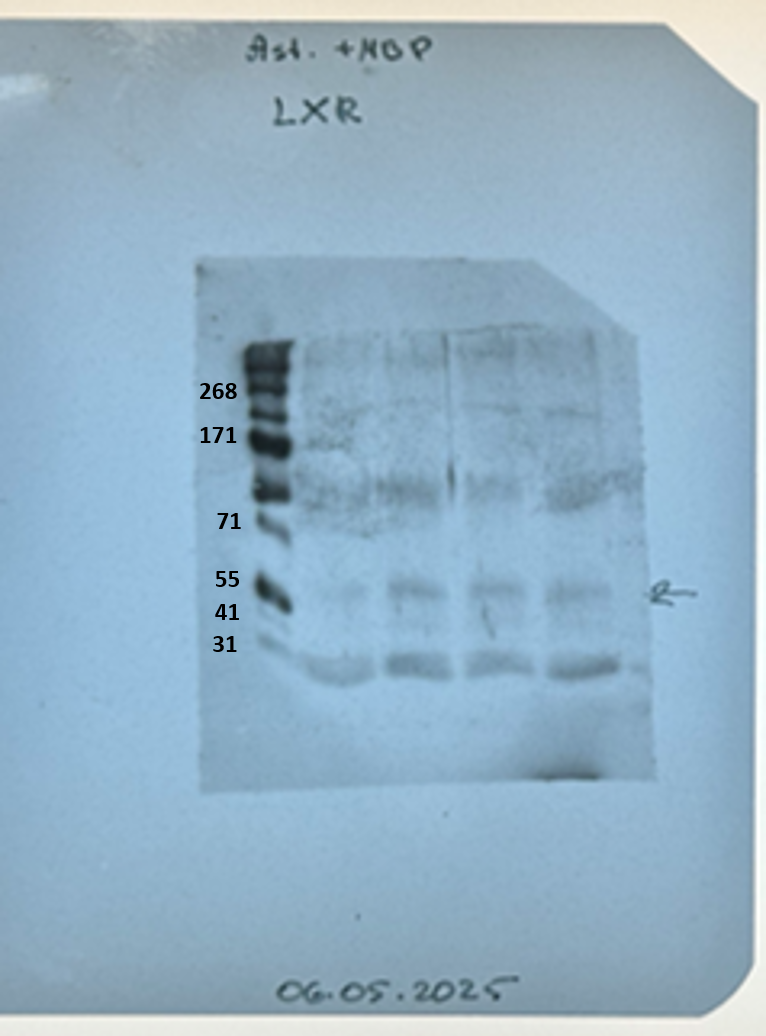

Supplement: Supplementary file 1 [file neurosci-07-00026-s001.zip › Figure S8 WB-Suppl.for Fig.8_.tif]

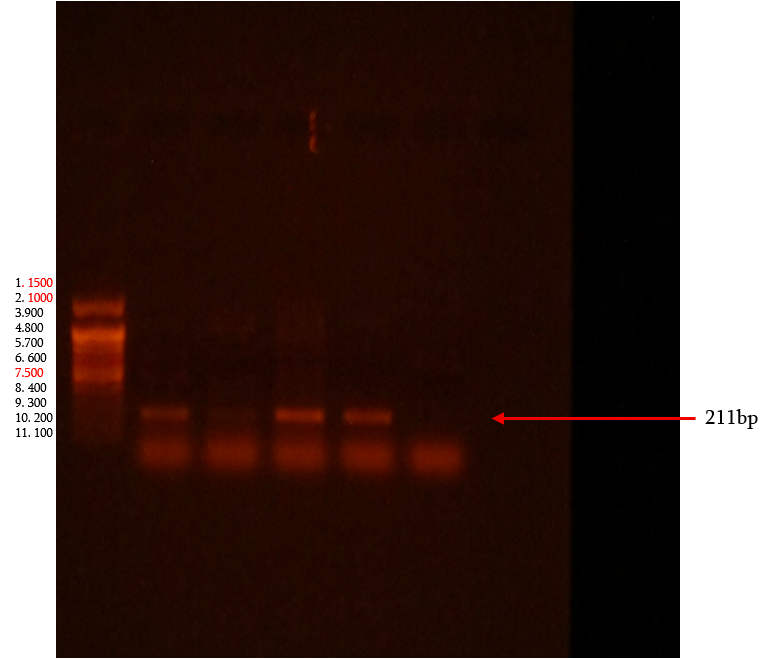

Supplement: Supplementary file 1 [file neurosci-07-00026-s001.zip › Figure S9 PCR-Suppl.for 9(3).tif]

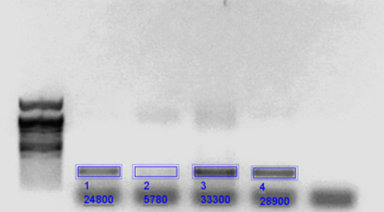

Supplement: Supplementary file 1 [file neurosci-07-00026-s001.zip › Figure S9 PCR-Suppl. for Fig.9(2).tif]

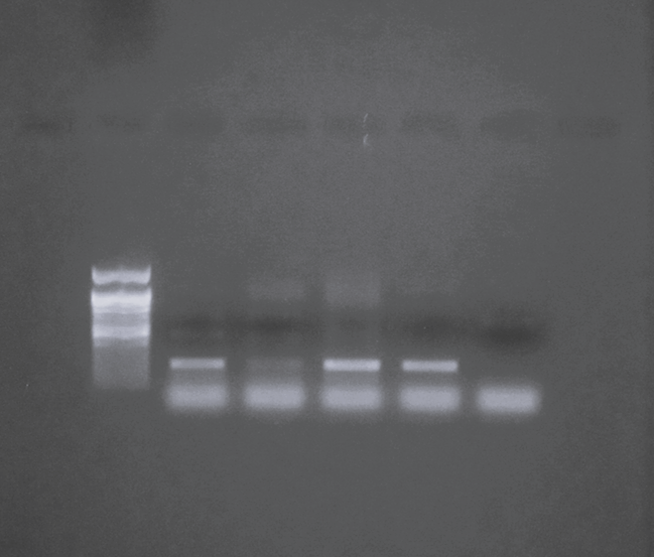

Supplement: Supplementary file 1 [file neurosci-07-00026-s001.zip › Figure S9 PCR-Suppl. for Fig9(1).tif]
